# The Mediating Effect of Physiological Health Fear Between Depressive Mood and Sleep Quality in Elderly Parkinson's Disease Patients

**DOI:** 10.1002/brb3.71670

**Published:** 2026-09-15

**Authors:** Ling Wu, Lihua Lu, Xiaopei Zhang, Tian Xu, Xiaosu Gu, Chen Shen

**Affiliations:** ^1^ Department of Neurology Affiliated Hospital of Nantong University Nantong Jiangsu China; ^2^ Department of Neurology School of Nursing and Rehabilitation,Nantong University Nantong Jiangsu China

**Keywords:** Parkinson's disease, depressive mood, sleep quality, fear of progression, physiological health fear, mediation analysis

## Abstract

**Objective:**

To investigate the relationship between depressive mood and sleep quality in elderly patients with Parkinson's disease (PD), and to analyze the mediating role of fear of progression (FoP).

**Methods:**

Between June 2024 and April 2025, 234 elderly patients with PD attending the Affiliated Hospital of Nantong University were enrolled. Participants completed the General Demographic Questionnaire, the 15‐item Geriatric Depression Scale (GDS‐15), the Fear of Progression Questionnaire Short Form (FoP‐Q‐SF), and the Parkinson's Disease Sleep Scale (PDSS). Spearman's rank correlation analysis was used to assess associations among variables. Mediation analyses were conducted using the PROCESS macro (version 4.0) in SPSS.

**Results:**

Sleep quality [median (IQR): 104 (84–130)] was significantly negatively correlated with depressive mood [7 (3–11)], total FoP [35 (30–40)], and the physiological health fear subscale [19 (17–22)] (*r* = −0.434, −0.318, −0.419, respectively; all *p* < 0.01), as well as with the social‐family fear subscale [16 (14–18)] (*r* = −0.168, *p* < 0.05). Depressive mood was positively correlated with total FoP, physiological health fear, and social‐family fear (*r* = 0.632, 0.675, 0.481, respectively; all *p* < 0.01). Mediation analysis revealed that depressive mood had a significant direct effect on sleep quality (*β* = −1.772, 95% CI: −2.839 to −0.706). Furthermore, the physiological health fear dimension of FoP partially mediated the relationship between depressive mood and sleep quality (indirect effect *β* = −1.010, 95% CI: −1.723 to −0.314).

**Conclusion:**

In elderly patients with PD, depressive mood directly impairs sleep quality and also exerts an indirect negative effect via fear related to physiological health deterioration. Targeting both depression and FoP, particularly physiological health concerns may improve sleep outcomes in this population.

## Abstract 2

This study examined whether fear of progression mediates the relationship between depressive mood and sleep quality in 211 elderly Parkinson's disease patients. Depressive mood directly impaired sleep quality (β = −1.772) and indirectly through physiological health fear (indirect effect β = −1.010, 95% CI: −1.723 to −0.314), accounting for 36.31% of the total effect. Findings suggest that targeting both depression and disease‐specific fearsparticularly concerns about physical deteriorationmay improve sleep outcomes in this population.

## 1 Introduction

Parkinson's disease (Parkinson's Disease, PD) is a disease whose main pathological feature is the progressive degeneration of dopaminergic neurons in the midbrain substantia nigra, and its incidence significantly increases with age (Liu et al. 2025). Although the typical clinical manifestations of PD are motor dysfunction, in recent years, the profound impact of non‐motor symptoms (Non‐Motor Symptoms, NMS) on patients’ overall health and quality of life has increasingly attracted attention (Janz et al. 2024); among NMS, sleep disorders and depressive mood are particularly prominent, and the two are interrelated, constituting a major clinical management challenge (Iranzo et al. 2024).

Studies show that the incidence of sleep disorders in PD patients remains persistently high, generally ranging from 47.66% to 89.10% (Iranzo et al. 2024; Yin et al. 2023). The manifestations of sleep disorders in Parkinson's disease are heterogeneous; commonly observed disturbances include insomnia, excessive daytime sleepiness (EDS), rapid eye movement (REM) sleep behavior disorder (RBD), periodic leg movements in sleep (PLMS), and restless legs syndrome (RLS), etc (Anderson et al. 2025). The decline in sleep quality in PD patients not only affects their daytime function and quality of life, but may also form a vicious cycle with the pathological progression of PD through multiple mechanisms (such as exacerbation of neuroinflammation, impairment of metabolic waste clearance), further worsening cognitive function and motor symptoms (Chen et al. 2025; Yin et al. 2023). Therefore, improving sleep quality in PD patients concerns not only symptom relief, but is also key to delaying disease progression and improving long‐term prognosis. The prevalence of depression in PD patients shows high heterogeneity, ranging from nearly 3% to as high as 90.0% (Qin et al. 2024). Multiple studies have confirmed that depression not only affects patients’ mental health status, but also has a close bidirectional association with sleep disorders (Ma et al. 2022); meanwhile, the synergistic effects between depression and other non‐motor symptoms—such as fatigue and pain—jointly exacerbate the disease burden on patients (Koh et al. 2022).

Fear of progression (FoP) has been widely studied in cancer and is increasingly applied to chronic diseases (Kenny‐Jones et al. 2024; Sharpe et al. 2023). A growing body of evidence has confirmed that FoP is not unique to cancer but represents a transdiagnostic construct across a broad range of chronic illnesses, including diabetes, rheumatoid arthritis, and multiple sclerosis (Menzies et al. 2025). FoP refers to patients’ persistent worry about disease deterioration and its potential adverse prognosis (such as functional loss, decline in self‐care ability), and has been confirmed to be significantly associated with negative emotions such as anxiety and depression, and may jointly impair patients’ sleep quality (Yun et al. 2025; Jrad et al. 2024). However, current research on the relationship among fear of progression, depressive mood, and sleep quality in PD patients remains relatively scarce. This study aims to explore the pathway and degree of influence of depressive mood on sleep quality in elderly PD patients, and to analyze the mediating role of fear of progression (FoP) therein, in order to provide theoretical basis for clinical intervention, help improve sleep quality in elderly PD patients, and enhance long‐term disease management outcomes.

## 2 Subjects and Methods

### 2.1 Subjects

This study adopted a cross‐sectional research design, and recruited Parkinson's disease patients from the neurology outpatient clinic of the Affiliated Hospital of Nantong University between June 2024 and April 2025.

Inclusion criteria: (1) meets diagnostic criteria for Parkinson's disease; (2) age ≥60 years; (3) mentally alert, able to cooperate and complete the questionnaire survey; (4) voluntarily participates in this study and signs the informed consent form.

Exclusion criteria: (1) combined with other serious neurological diseases; (2) has severe cognitive impairment and is unable to understand questionnaire content; (3) has a history of mental illness; (4) used medications within the past month that significantly affect mood or sleep, etc.

A total of 234 questionnaires were distributed, of which 211 were valid, with an effective recovery rate of 90.2%.

## 3 Methods

### 3.1 Research Instruments

1. General Demographic Scale: Self‐designed, including basic information of patients such as gender, age, education level, place of residence, income level, disease duration, Hoehn & Yahr stage, etc.
2. The Chinese version of the Fear of Progression Questionnaire‐Short Form (FoP‐Q‐SF) was used to assess patients’ fear of disease progression. This scale originated in Germany and was simplified by Mehnert et al. (2006); it covers two dimensions, physiological health fear and social‐family fear, with 6 items per dimension (using a Likert 5‐point rating scale). A total score ≥34 points indicates that the patient's fear of disease progression is at a high level. The Cronbach's α coefficient of the Chinese version of the scale is 0.883 (Qiyun et al. 2015); in this study, the Cronbach's α coefficient was 0.899.
3. The Parkinson's Disease Sleep Scale (PDSS) was used to quantitatively assess sleep quality in PD patients. This scale was developed by Chaudhuri et al. (2002) in 2002 and has demonstrated high sensitivity and reliability. At the time of study initiation (early 2024), the Chinese version of the original PDSS had been well established and widely adopted in clinical research in China, whereas the PDSS‐2 had not yet been formally validated for use in the Chinese population. Therefore, the original PDSS was chosen as the most psychometrically sound and culturally appropriate instrument for this study. It comprises 15 items rated on a 0–10 visual analogue scale, covering nocturnal sleep quality overall, rapid‐eye‐movement sleep behaviour disorder, insomnia, nocturnal motor symptoms, nocturia, and daytime somnolence. The total score ranges from 0 to 150; the lower the score, the worse the sleep quality; a total score ≤90 points indicates the presence of sleep disturbance. In this study, the Cronbach's α coefficient of this scale was 0.813.
4. The short‐form Geriatric Depression Scale (GDS‐15) was used to screen for depressive mood in elderly PD patients (Sheikh et al. 1988; Shin et al. 2019). The scale contains 15 questions, each answered with “yes” or “no,” with a scoring range of 0–15 points; a score >5 points indicates presence of depressive mood, and the higher the score, the more severe the depressive symptoms. In this study, the Cronbach's α coefficient of this scale was 0.899.

### 3.2 Data Collection Method

Trained researchers administered all questionnaires in person. Prior to participation, written informed consent was obtained from all participants. For individuals with limited literacy, researchers provided standardized verbal instructions and assisted with questionnaire completion to ensure accurate responses. All questionnaires were collected on‐site immediately after completion. Incomplete or inconsistent responses were excluded from analysis. To ensure data accuracy, dual independent data entry was performed, followed by cross‐verification to resolve discrepancies.

### 3.3 Statistical Methods

Statistical analysis was performed using SPSS 26.0 software. Fear of progression scores, sleep quality scores, and depression scores were all skewed continuous variables, described using M (Q1, Q3). Spearman's correlation analysis was used to examine correlations. A structural equation model was used to test the mediating role of fear of progression between depressive mood and sleep quality. Gender, age, education level, and other variables were entered into the equation as covariates. Confidence interval is 95%; bootstrap method (5000 resamples) was used to test the mediating effect. *p* < 0.05 was considered statistically significant.

## 4 Results

### 4.1 Common Method Bias Test

Common method bias was assessed using Harman's single‐factor test. Exploratory factor analysis (EFA) without rotation yielded nine factors with eigenvalues greater than 1, cumulatively explaining 65.87% of the variance. The first unrotated factor accounted for 27.71% of the variance, well below the recommended 40% threshold, suggesting that common method bias is not a significant concern in this study.

### 4.2 General Characteristics

Among the 211 elderly patients with Parkinson's disease, 126 (59.72 %) were male and 85 (40.28 %) were female; the median age was 73 years (IQR: 68–77). Details are presented in Table 1.

**TABLE 1:** General information of elderly patients with Parkinson's disease (*n* = 211).

Item	*N*	Percentage(%)	Item	*n*	Percentage(%)
Gender			Medication insurance		
Male	126	59.7	Resident basic medical insurance	142	67.3
Female	85	40.3	Employee basic medical insurance	69	32.7
Age (years)			Disease duration (years)		
60–69	65	30.8	<1	28	13.3
70–79	125	59.2	1–5	94	44.5
≥80	21	10.0	≥5	89	42.2
Education level			Number of comorbid chronic diseases		
Primary school or below	130	61.6	0	76	36.0
Junior high school	62	29.4	1	87	41.3
Senior high school or above	19	9.0	2	41	19.4
Marital status			≥3	7	3.3
Married	173	82.0	Self‐care ability		
Unmarried	38	18.0	Fully independent	94	44.5
Residence	173	82.0	Partially dependent	104	49.3
Urban	118	55.9	Fully dependent	13	6.2
Rural	93	44.1	Exercise Habit		
Monthly income (CNY)			Rarely	160	75.8
<1000	85	40.3	Occasionally	41	19.4
1000–3000	51	24.2	Regularly	10	4.8
>3000	75	35.5	Constipation		
Smoking			Yes	156	73.9
Yes	31	14.7	No	55	26.1
No	180	85.3	Hoehn & Yahr stage		
Drinking			Early	109	51.7
Yes	31	14.7	Middle	69	32.7
No	180	85.3	Late	33	15.6

#### 4.2.1 Differences in Fear of Progression, Depression, and Sleep Quality Across General Characteristics in Elderly Patients With Parkinson's Disease

Differences in fear of progression, depression, and sleep quality across baseline characteristics among elderly patients with Parkinson's disease are detailed in Table 2. Statistically significant differences were observed with respect to disease duration, self‐care ability, exercise habit, constipation, and Hoehn & Yahr stage (all *p* < 0.01).

**TABLE 2:** Differences in fear of progression, depression, and sleep quality across general characteristics in elderly patients with Parkinson's disease (*n* = 211).

Item	FoP‐Q‐SF		GDS‐15		PDSS	
	*F*/*t*	*p*	*F*/*t*	*p*	*F*/*t*	*p*
Gender	2.446	0.015	1.97	0.05	−0.197	0.844
Age	0.579	0.563	0.986	0.325	−2.52	0.012
Disease duration	3.136	0.002	4.491	<0.001	−3.917	<0.001
Self‐care ability	8.07	<0.001	9.171	<0.001	−6.954	<0.001
Exercise habit	−3.162	0.002	−4.741	<0.001	4.362	<0.001
Constipation	4.192	<0.001	5.123	<0.001	−4.589	<0.001
Hoehn & Yahr stage	5.849	<0.001	7.538	<0.001	−6.326	<0.001

#### 4.2.2 Fear of Progression, Sleep Quality, and Depression Scores in Elderly PD Patients, and Correlation Analysis

Among the 211 elderly patients with Parkinson's disease, median scores were as follows: depressive mood, 7 (IQR: 3–11); fear of progression (FoP), 35 (30–40); physiological health fear, 19 (17–22); social‐family fear, 16 (14–18); and sleep quality, 104 (84–130). Spearman's correlation analysis revealed that sleep quality was significantly negatively correlated with depressive mood (*r* = −0.434, *p* < 0.01), total FoP (*r* = −0.318, *p* < 0.01), and physiological health fear (*r* = −0.419, *p* < 0.01). Weaker but still significant negative correlations were observed between sleep quality and both age (*r* = −0.159, *p* < 0.05) and social‐family fear (*r* = −0.168, *p* < 0.05). Depressive mood was positively correlated with total FoP (*r* = 0.632, *p* < 0.01), physiological health fear (*r* = 0.675, *p* < 0.01), and social‐family fear (*r* = 0.481, *p* < 0.01). Detailed correlation coefficients and significance levels are presented in Table 3.

**TABLE 3:** Correlation analysis of fear of progression, depression, and sleep quality in Parkinson's disease patients (*n* = 211).

Item	M(Q25,Q75)	Age	FoP‐Q‐SF	Physiological health fear	Social‐family fear	PDSS	GDS‐15
Age	73 (68, 77)	1					
FoP‐Q‐SF	35 (30, 40)	−0.025	1				
Physiological health fear	19 (17, 22)	0.003	0.912^b^	1			
Social‐family fear	16 (14, 18)	−0.083	0.915^b^	0.718^b^	1		
PDSS	104 (84, 130)	−0.159^a^	−0.318^b^	−0.419^b^	−0.168^a^	1	
GDS‐15	7 (3, 11)	0.050	0.632^b^	0.675^b^	0.481^b^	−0.434^b^	1

FoP‐Q‐SF, Fear of Progression Questionnaire‐Short Form; PDSS, Parkinson's Disease Sleep Scale; GDS‐15, Geriatric Depression Scale.

^a^ *p* < 0.05.

^b^ *p* < 0.01.

#### 4.2.3 Mediating Role of Fear of Progression Between Depressive Mood and Sleep Quality

After controlling for demographic covariates (gender, age, and education level), we examined the mediating roles of fear of progression (FoP) and its subscales in the relationship between depressive mood and sleep quality.

In the mediation models “Depressive Mood → Total FoP → Sleep Quality” and “Depressive Mood → Social‐Family Fear → Sleep Quality,” the indirect effects were not statistically significant:

- FoP → Sleep Quality: *β* = −0.286, 95% CI [−0.890, 0.319], *p* = 0.352
- Social‐Family Fear → Sleep Quality: *β* = 0.303, 95% CI [−0.706, 1.311], *p* = 0.556

Since both confidence intervals included zero and *p* > 0.05, these pathways were not pursued further.
In contrast, the pathway “Depressive Mood → Physiological Health Fear → Sleep Quality” yielded statistically significant direct and indirect effects:

- Direct effect: *β* = −1.772, 95% CI [−2.839, −0.706], *p* < 0.01
- Indirect effect: *β* = −1.010, 95% CI [−1.723, −0.314], *p* < 0.01

**FIGURE 1:**
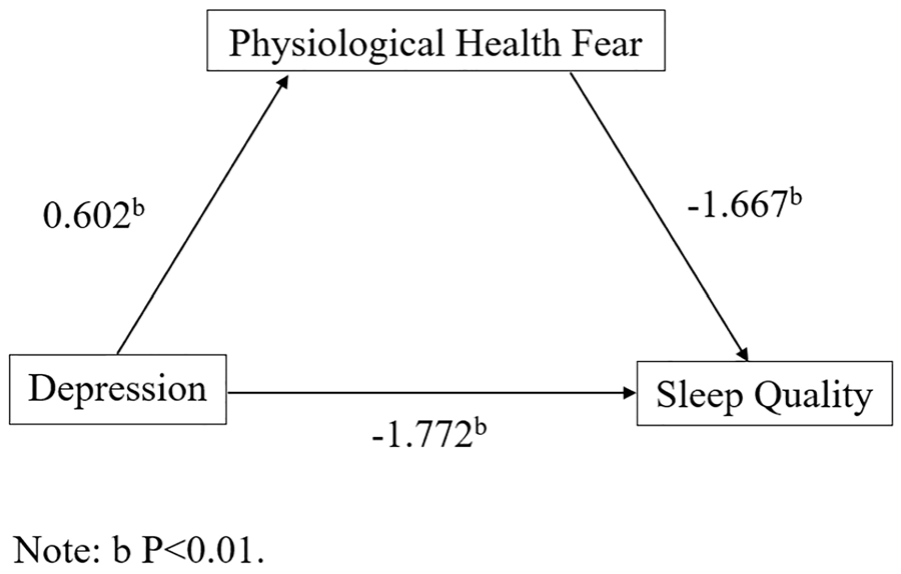
Mediation model of physiological health fear between depressive and sleep quality in elderly Parkinson's disease patients. Values shown are standardized regression coefficients. ^b^ *p* < 0.01. The direct effect of depressive mood on sleep quality (*β* = –1.772, 95% CI: –2.839 to –0.706) and the indirect effect via physiological health fear (*β* = –1.010, 95% CI: –1.723 to –0.314) are presented. The physiological health fear dimension accounted for 36.31% of the total effect.

The proportion of the total effect mediated by physiological health fear was 36.31%, indicating a partial mediation. These results suggest that physiological health‐related fear significantly mediates the association between depressive mood and sleep quality in elderly PD patients.
Detailed results are presented in Tables 4 and 5 and illustrated in Figure 1.

**TABLE 4:** Mediation effect analysis and testing between depression and sleep quality in Parkinson's disease patients.

Serial number	Model	*R* ^2^	*F* value	*β* value	*t* value	Standard error	95% CI	*p*
1	Depression → FoP‐Q‐SF → sleep quality	0.207	13.445	−2.782	−6.702	0.415	0.901 to 1.273	<0.001
	Depression → FoP‐Q‐SF			1.087	11.528	0.094	−3.600 to –1.964	<0.001
	FoP‐Q‐SF → sleep quality			−0.286	−0.931	0.307	−0.890 to 0.319	0.352
	Depression → sleep quality			−2.471	−4.640	0.533	−3.521 to –1.421	<0.001
2	Depression → physiological health fear → sleep quality	0.207	13.445	−2.782	−6.702	0.415	−3.600 to –1.963	<0.001
	Depression → physiological health fear			0.602	12.486	0.048	0.507 to 0.697	<0.001
	Physiological health fear → sleep quality			−1.677	−2.843	0.590	−2.840 to –0.514	<0.005
	Depression → sleep quality			−1.772	−3.276	0.541	−2.839 to –0.706	0.001
3	Depression → social‐family fear → sleep quality	0.207	13.445	−2.782	−6.701	0.415	−3.600 to –1.963	<0.001
	Depression → social‐family fear			0.475	8.388	0.057	0.364 to 0.587	<0.001
	Social‐family fear → sleep quality			0.302	0.590	0.511	−0.706 to 1.311	0.556
	Depression → sleep quality			−2.925	−6.075	0.482	−3.875 to –1.976	<0.001

*Note*: Gender, age, education level, and other variables were entered into the equation as covariates.

**TABLE 5:** Mediating effect of physiological health fear between depression and sleep quality in Parkinson's disease patients.

Item	Effect value	Standard error	95% CI	Effect proportion (%)
Total effect	−2.782	0.415	−3.600 to –1.963	100
Direct effect	−1.772	0.541	−2.839 to –0.706	63.69
Indirect effect	−1.010	0.356	−1.723 to –0.314	36.31

## 5 Discussion

Survey results revealed a median sleep quality score of 104 (IQR: 84–130), with 62.09% of participants reporting clinically significant sleep disturbances—consistent with the high prevalence rates reported in prior studies (Iranzo et al. 2024). Among neurodegenerative disorders, sleep disturbances in Parkinson's disease (PD) are more prevalent than in Alzheimer's disease (Sondell et al. 2021) or Huntington's disease (Shen et al. 2023). These disturbances extend beyond insomnia or nocturnal awakenings to include profound disruptions in sleep architecture (Yin et al. 2023), and are closely intertwined with psychological state and social environment, forming a self‐perpetuating mind‐body cycle.

The median depressive mood score was 7 (IQR: 3–11), with a prevalence of 56.87%—significantly higher than that observed in the general elderly population (Liu et al. 2023). This elevated rate likely reflects not only psychological distress but also the neurobiological consequences of PD, particularly degeneration along the dopaminergic‐limbic pathways (Cong et al. 2022). Additionally, the functional limitations and increased dependency imposed by PD may further heighten vulnerability to depression.

Fear of progression (FoP) was also prominent, with a median total score of 35 (IQR: 30–40). Subscale scores revealed higher levels of physiological health fear (median = 19, IQR: 17–22) compared to social‐family fear (median = 16, IQR: 14–18). These scores exceed that reported in diabetes patients (Wang et al. 2022), likely due to the unpredictable nature of PD motor fluctuations (e.g., “on‐off” phenomena) and diminishing medication efficacy, which foster a sense of uncontrollability. Although dopamine agonists may alleviate motor symptoms, they can also induce impulse control disorders (ICDs), potentially exacerbating fears of behavioral dyscontrol (Cuoco et al. 2025). The predominance of physiological health fear over social‐family fear may reflect the advanced age of participants, for whom physical health concerns often take precedence over social or familial worries. This pattern aligns with the broader literature on fear of progression as a transdiagnostic construct across chronic illnesses, where the salience of specific fear domains often varies according to disease characteristics, symptom burden, and individual contextual factors (Sharpe et al. 2023).

The study results indicate that depressive mood in PD patients is significantly negatively correlated with sleep quality—that is, the more severe the depressive symptoms, the poorer the sleep quality, consistent with previous findings (Mayeli et al. 2024). As a persistent negative emotional state, depression can activate the body's stress response system, particularly the hypothalamic‐pituitary‐adrenal (HPA) axis, leading to elevated cortisol levels that disrupt the normal circadian secretion of sleep‐regulating hormones such as melatonin (Cheng et al. 2021), thereby contributing to difficulties in sleep onset and increased vulnerability to nocturnal awakenings and fragmented sleep. In this study, the total FoP‐Q‐SF score and its subscales—especially physiological health fear—were significantly positively correlated with depressive mood and negatively correlated with sleep quality, aligning with the results reported by Yun et al. (Yun et al. 2025). Among PD patients, depressive mood does not exist in isolation but interacts dynamically with motor symptoms—for example, bradykinesia and tremor may worsen during depressive episodes, which in turn amplifies patients’ fears regarding their deteriorating physical condition, creating a self‐reinforcing cycle wherein psychological distress, physiological fear, and disrupted sleep mutually exacerbate one another, ultimately resulting in a clinically significant decline in overall sleep quality (Wang et al. 2024; Lacy et al. 2022).

Although prior studies have established a significant association between depressive mood and sleep disturbance, exploration of the complex mediating pathways underlying this relationship remains limited. This study, through structural equation modeling and mediation analysis, is the first to demonstrate that the physiological health fear dimension of fear of progression (FoP) plays a key mediating role in the link between depressive mood and sleep quality in elderly PD patients. Results show that physiological health fear exerts a significant partial mediation effect (*β* = −1.010, 95% CI [−1.723, −0.314]), accounting for 36.31% of the total effect (*p* < 0.001). This finding, interpreted through the lens of cognitive‐stress theory, suggests that negative emotions may disrupt sleep homeostasis via dual pathways: illness‐related cognitive biases and sustained psychological stress responses (Agnoli et al. 2024). Specifically, a depressive state may amplify patients’ catastrophic appraisals of physiological decline, perpetually activating the hypothalamic‐pituitary‐adrenal (HPA) axis and maintaining elevated cortisol levels—which not only disturb circadian sleep rhythms but may also accelerate neurodegenerative processes in PD, thereby reinforcing a self‐sustaining “emotion–cognition–physiology” vicious cycle (Knezevic et al. 2023; Luthra et al. 2023). These results highlight that patients’ fear of disease‐related bodily deterioration is a critical psychological mechanism bridging depression and sleep disruption, offering a novel conceptual framework for understanding psychophysiological interactions in PD and informing future targeted interventions.

However, in the two mediation pathways: “Depressive Mood → Total FoP → Sleep Quality” and “Depressive Mood → Social‐Family Fear → Sleep Quality”—the 95% confidence intervals for the indirect effects each included zero, indicating no statistically significant mediation. This may reflect the fact that the overall influence of fear of progression is moderated by multiple contextual and psychological factors; although the social‐family fear dimension is closely tied to patients’ interpersonal and domestic environments, it is possible that robust family support systems buffered the impact of depressive mood on this specific fear, preventing it from translating into a measurable decline in sleep quality. This finding underscores the complexity of psychosocial pathways in PD and suggests that future research should further investigate other potential moderators (e.g., social support, coping style, disease duration) and mechanisms linking FoP to sleep disturbance, as well as examine heterogeneous effects across subgroups of patients (e.g., by age, care setting, or comorbidity profile).

The results of this study have direct guiding value for the comprehensive management of elderly PD patients. Given the significant presence of the mediating effect of the physiological health fear dimension, clinical interventions may attempt to start by interrupting the negative cognitive cycle, focusing on correcting patients’ catastrophic cognitions regarding disease progression and physical function. Specific strategies may include: (1) tiered disease knowledge education based on individual patient conditions, helping patients establish a scientifically rational cognitive framework of disease progression; (2) using cognitive behavioral therapy (CBT) to guide patients in reconstructing catastrophic thinking patterns regarding symptom changes, thereby reducing levels of excessive fear (Höglinger et al. 2024); (3) supplementing with personalized sleep hygiene guidance and physiological feedback relaxation training, to improve sleep quality through multiple dimensions.

## 6 Methodological Considerations and Limitations

A key methodological consideration in this study is the absence of a sex‑ and age‑matched healthy control group. This design choice was intentional and grounded in the primary objective of our investigation: to elucidate the within‑group mediating pathway linking depressive mood to sleep quality in elderly Parkinson's disease (PD) patients. For mediation analysis, the statistical power to detect indirect effects is primarily contingent upon the variance of the exposure, mediator, and outcome variables within the sample, rather than on comparison with a non‑exposed reference group (Lee et al. 2024). Thus, a cross‑sectional single‑group design is methodologically appropriate for examining mechanistic associations, provided the sample demonstrates sufficient variability in the key constructs. Our cohort exhibited substantial heterogeneity in disease duration, Hoehn & Yahr stage, self‑care ability, exercise habits, constipation, age, and gender, which collectively ensured adequate variance to test the hypothesized mediation pathway.

Nevertheless, we fully acknowledge that the absence of a control group, along with other design limitations, warrants caution in interpreting our findings. Both sleep disturbances and depressive mood are highly prevalent in the general older population (Zhang et al. 2023), and without a matched control group, we cannot definitively determine whether the identified mediation pathway is specific to PD pathology or reflects more general psychosomatic processes associated with aging and chronic illness. Furthermore, we acknowledge that clinically relevant phenotypic characteristics—such as motor subtype and REM sleep behavior disorder (RBD) status—were not systematically incorporated into the primary analysis. The absence of detailed phenotypic stratification limits our ability to determine whether the observed mediating effect is uniformly applicable across all clinical subtypes or is particularly salient in specific subgroups, such as those with greater axial impairment or more prominent RBD‑related disturbances. Future studies with larger, comprehensively phenotyped samples should conduct stratified or interaction analyses to address this gap.

Several additional limitations warrant consideration. First, the cross‑sectional design precludes the establishment of strict temporal causality among depression, fear of progression, and sleep quality. Second, the sample was drawn exclusively from a single neurology outpatient clinic in eastern China, which may limit generalizability. Third, while the PDSS is a well‑validated screening tool, it provides only a global assessment of sleep quality and does not comprehensively capture the full spectrum of sleep architecture disturbances. Moreover, the original PDSS was used rather than the updated PDSS‑2, which offers a more detailed frequency‑based assessment and better differentiation of nocturnal motor symptoms. However, at the time of study initiation (early 2024), the PDSS‑2 had not yet been formally validated for use in the Chinese population, whereas the original PDSS had been well established and widely adopted. Future studies should consider using PDSS‑2 when the validated Chinese version becomes widely accessible. Fourth, detailed information on medication use—including antiparkinsonian drug dosages, antidepressants, and anxiolytics—was not collected. Although we excluded patients who had used medications significantly affecting mood or sleep within the past month to minimize short‑term confounding, long‑term medication regimens may still influence the observed associations. Fifth, data on participants' living arrangements and social connectedness were not systematically collected, despite both being known to influence depressive symptoms and sleep quality in older adults.

In summary, while our findings contribute novel evidence for the mediating role of physiological health fear in the depression‑sleep relationship among elderly PD patients, the above methodological constraints should be considered when interpreting the results. Future research should employ longitudinal designs, incorporate healthy control groups, expand sampling to multi‑center and community‑based settings, utilize more comprehensive sleep assessments, conduct stratified analyses by clinical phenotype, and systematically collect medication and social support data to further validate and extend our findings.

## 7 Conclusion

In summary, elderly patients with Parkinson‘s disease in this cohort exhibited relatively high levels of depressive mood and fear of progression, and generally reported poor sleep quality. Depressive mood not only directly impaired sleep quality but also exerted an indirect negative effect through the physiological health‐related dimension of fear of progression. These findings suggest that the physiological health fear dimension, rather than the social‐family fear dimension, is the key psychological mechanism linking depression to sleep disruption in elderly PD patients. This study contributes novel evidence for the mediating role of physiological health fear and provides a theoretical foundation for developing targeted psychological interventions that address both depressive symptoms and disease‐specific fears—particularly concerns about physical deterioration—to improve sleep outcomes and overall well‐being in this vulnerable population.

## Author Contributions

**Ling Wu**: Data curation, Formal Analysis, Methodology, Software, Writing – original draft. **Lihua Lu**: Data curation, Software, Writing – original draft. **Xiaopei Zhang** and **Tian Xu**: Formal Analysis, Methodology, Writing – original draft. **Xiaosu Gu** and **Chen Shen**: Conceptualization, Supervision, Validation, Visualization, Writing – review & editing. All authors contributed to the manuscript and approved the final version for submission.

## Funding

This is is funded by Jiangsu Province Research‐Oriented Hospital Project (Grant No. YJXYY202204).

## Conflicts of Interest

The authors declare that they have no conflict of interest.

## Ethics Statement

This paper was approved by Ethics Committee of Affiliated Hospital of Nantong University (number: 2023‐K014‐01), and we were in accordance with the 1975 Helsinki declaration and its later amendments.

## Generative AI Disclosure

This article was written by the author independently, without using any AI tools or software to generate, edit, or modify the content.

## Data Availability

All data generated or analyzed during this study are included in this published article.
